# The trafficking of Na_V_1.8

**DOI:** 10.1016/j.neulet.2010.08.074

**Published:** 2010-12-10

**Authors:** Richard S. Swanwick, Alessandro Pristerá, Kenji Okuse

**Affiliations:** Division of Cell & Molecular Biology, Imperial College London, London SW7 2AZ, UK

**Keywords:** Sodium Channel, Sensory Neuron, Pain, Trafficking

## Abstract

The α-subunit of tetrodotoxin-resistant voltage-gated sodium channel Na_V_1.8 is selectively expressed in sensory neurons. It has been reported that Na_V_1.8 is involved in the transmission of nociceptive information from sensory neurons to the central nervous system in nociceptive [1] and neuropathic [24] pain conditions. Thus Na_V_1.8 has been a promising target to treat chronic pain. Here we discuss the recent advances in the study of trafficking mechanism of Na_V_1.8. These pieces of information are particularly important as such trafficking machinery could be new targets for painkillers.

## Research highlights

▶ The β3 subunit masks the ER retention signal of Na_V_1.8 and release the channel from the ER. ▶ p11 directly binds to Na_V_1.8 and help its translocation to the plasma membrane. ▶ PDZD2 is responsible for the functional expression of Na_V_1.8 on the plasma membrane. ▶ Contactin KO mice exhibit a reduction of Na_V_1.8 along unmyelinated axons in the sciatic nerve. ▶ PKA activation increases the Na_V_1.8 density on the membrane through direct phosphorylation.

Membrane proteins are translated in the rough ER, post-translationally modified and sorted during their migration through the Golgi to the plasma membrane. Interestingly, a large sub-population of voltage-gated sodium channels are not directly translocated to the plasma membrane, but stored in a metabolically stable ‘intracelluar pool’ of folded protein [36]. This pool may permit the neuron to respond rapidly to stimuli, allowing it to increase channel surface density faster than *de novo* synthesis would otherwise allow. Recent reports describe many novel mechanisms which regulate the trafficking of ion channels to the plasma membrane occurring at many points along the pathway including the initial translation and folding, subsequent post-translational modification e.g. N-glycosylation and palmitylation and by numerous protein–protein interactions.

## 1 ER retention signals

**Fig. 1:**
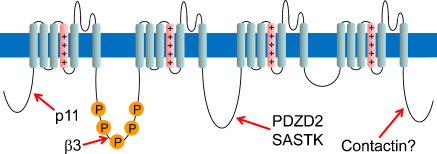
Structure of the Na_V_1.8 showing the four homologous domains, each of which is composed of six membrane spanning segments. The S4 voltage sensors are shown in pink. The binding sites for the Na_V_1.8-interacting proteins are shown. The five sites for PKA phosphorylation at serine residues are indicated as “P”.

The RXR motif, which serves as an ER retention signal, is found in many membrane proteins, including ATP-sensitive potassium channels [53], NMDA [40] and GABAB receptors [12]. Na_V_1.8 possesses at least one functional ER retention signal within the first intracellular loop, consisting of residues RRR495–497 [54]. Substitution of this motif with alanines results in an increased expression of the channel on the membrane. The mechanism behind Na_V_1.8 release from the ER and trafficking to the cell surface has been described. The auxiliary β3 subunit, which is expressed in dorsal root ganglia (DRG) neurons, associates with the α-subunit of voltage-gated sodium channels [48], and plays a key role in this process. The intracellular C-terminus of the β3 subunit directly binds the portion of Na_V_1.8 containing the RRR signal [54] (Fig. 1). This interaction masks the retention signal and leads to the release of the channel from the ER. Other sodium channels also contain potential ER retention signals, although the position of this motif is not conserved (e.g. Na_V_1.5 – RKR amino acid 480–482). Hence, similar mechanisms to control retention/trafficking to the membrane could exist for the other sodium channel isoforms. It is known the β3 subunit is up-regulated in small diameter neurons upon nerve axotomy [48], chronic constriction injury [41] and, in medium size neurons, in diabetic neuropathy models [42]. Thus, the β3 subunit mediated Na_V_1.8 release from the ER could also account for increased excitability in pain states due to additional Na_V_1.8 being transported onto the cell membrane.

## 2 p11

Although the β3 subunit has been shown to help translocation of Na_V_1.8 from ER to the plasma membrane as described above, we found that co-expression of accessory β-subunits did not help the functional Na_V_1.8 expression in heterologous cells such as COS [3]. This suggested that Na_V_1.8 requires other accessory proteins for its functional expression on the plasma membrane and led to the discovery of p11 as a novel permissive factor for Na_V_1.8 [32]. p11 (S100A10, Annexin 2 light chain) is a member of the S100 calcium binding protein family, which regulates many cellular processes in response to intracellular calcium changes. p11 is expressed in a large number of tissues and is present in many regions of the CNS including the cerebral cortex, hippocampus and hypothalamus [38,55]. It exists as a tight, non-covalent dimer and is the only member of family to have suffered mutations within its EF hand motifs, rendering it Ca^2+^ insensitive [19]. Although p11 cannot respond to calcium fluctuations, it exists in a permanently “activated state” compared to other members of the S100 family [18]. The majority of p11 present intracellularly, exists as a heterotetramer (A2t), composed of two identical annexin II heavy chains [19]. Annexin II, interacts in a Ca^2+^-dependent manner with negatively charged phospholipids, also binding to cholesterol and many protein ligands including actin, and is thought to be involved in many membrane-related events including endosome membrane trafficking along the recycling and degradation pathways [23,2]. The phosphorylation by PKC of Ser11 of Annexin II disrupts p11 binding [20], while the activation of a protein phosphatase by PKA and subsequent dephosphorylation of the same residue [34] may allow coupling of the p11 localisation to the Ca^2+^ and cAMP-dependent cellular signalling pathways.

**Fig. 2:**
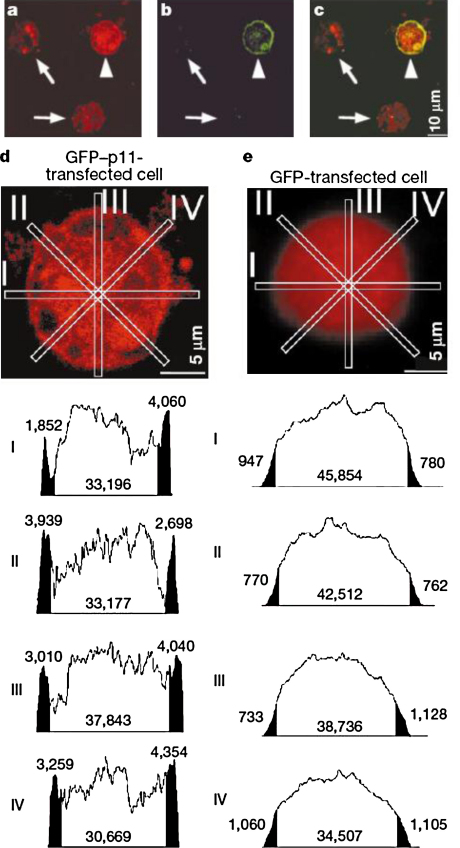
(a) Na_V_1.8-like immunoreactivity in CHO-SNS22 cells. (b) Transient expression of GFP-p11 fusion protein in CHO-SNS22 cell. (c) Merged picture of (a) and (b) showing co-expression of p11 and Na_V_1.8 in the plasma membrane. (d and e) Microscopic images were quantitated using the NIH Image program. Densitometric measurements show the clear translocation of Na_V_1.8 into the plasma membrane in GFP-p11 transfected cells.

Both yeast two-hybrid and GST pull-down assays demonstrate that p11 binds to the amino terminus of Na_V_1.8, especially resides 74–103, located close to the start of the initial transmembrane domain [30] (Fig. 1). Upon co-expression of p11 with Na_V_1.8 in heterologous cells such as CHO, the channel successfully translocated to the plasma membrane [32] (Fig. 2), even in the absence of additional β-subunits. In DRGs, in the absence of p11 (DRG specific knock-out), poor functional Na_V_1.8 expression is observed [9]. Interestingly, since functional expression is not totally abolished in the knock-out, additional factors expressed specifically by neurons, may be capable of trafficking a small amount of the channel. Significantly, p11 binds only to Na_V_1.8 and not to the other sodium channel subtypes also expressed in nociceptive neurons, such as Na_V_1.7 and Na_V_1.9 [33].

The identification of p11 as a chaperone protein for Na_V_1.8 has been followed by discoveries of many other membrane proteins controlled by p11 including TASK-1, 5-HT_1B_ receptors, ASIC-1, TrpV5, and TrpV6. p11 promotes the membrane localisation of TASK-1 by masking an ER retention signal at the end of the C-terminal of the channel [13]. Interestingly p11 has also been reported to act as an ER retention factor by binding to the other region of the C-terminal of TASK-1 [35]. At present the ability of p11 to act either ER retention factor or translocation promoter with different proteins is not understood. There is evidence to suggest that the carboxy terminus of p11 itself, may contain an ER retention motif, and thus interaction with other proteins may subsequently lead to ER retention [35]. Additionally, p11 could itself interact with other proteins masking their ER retention motifs, blocking their effect and subsequently releasing the protein from the ER.

## 3 PDZD2

**Fig. 3:**
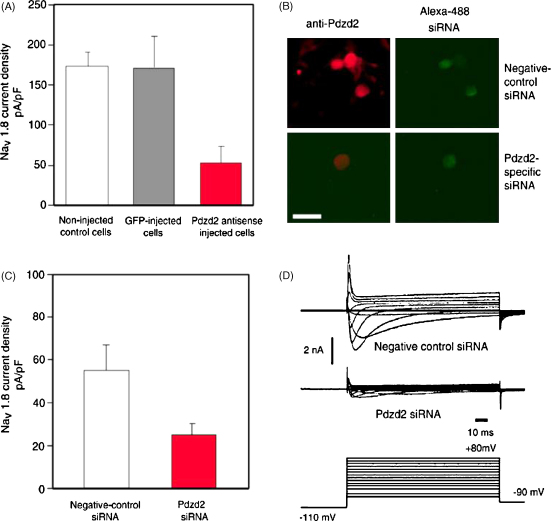
(a) PDZD2 antisense mRNA expression in DRG neurons caused a loss of Na_V_1.8 current density. (b) Transfection of PDZD2-specific siRNA (conjugated to Alexa Fluor-488) into cultured DRG neurons caused efficient and specific down-regulation of endogenous PDZD2 protein expression. (c) The transfection of PDZD2 siRNA caused significant reduction of the Na_V_1.8 current density in DRG neurons. (d) Example TTX-resistant inward current traces from negative control siRNA and PDZD2 siRNA treated DRG neurons.

PDZD2, also known as Papin, AIPC and PDZK3, is a large protein (2766 amino acid), which was identified as an interactor of Na_V_1.8 using the yeast two-hybrid system [30]. PDZD2 contains six PDZ domains, four located at the N-terminus and two at the C-terminus, and is thought to act as a scaffolding protein assisting the trafficking and/or clustering of target proteins [22]. PDZD2 is expressed in several tissues including heart, lung, pancreas and DRG [52]. In DRGs, PDZD2 is expressed in both small peripherin positive and large NF200 positive neurons [44]. PDZD2 has been shown to directly bind the second intracellular loop of Na_V_1.8 (between domain II and III) through its C-terminus (Fig. 1). Antisense mRNA and siRNA mediated knock-down of PDZD2 in DRG neurons *in vitro* leads to a drastic reduction of Na_V_1.8 mediated sodium currents [44] (Fig. 3). Whether PDZD2 regulates the trafficking or the retention of Na_V_1.8 within the membrane has not yet been addressed. PDZD2 has also been shown to bind Na_V_1.7 and, may bind to all voltage-gated sodium channels via conserved PDZ binding motifs [44,45]. The binding of PDZD2 to Na_V_1.8 and Na_V_1.7 (which together underlie action potential generation and propagation in unmyelinated nociceptive fibres) suggests that PDZD2 may modulate cell excitability and ultimately pain thresholds. The role of PDZD2 in pain sensitivity has been investigated in PDZD2 knock-out mice, however no alterations in pain behaviours were observed (mechanical, thermal and inflammatory pain models) [44]. The absence of a pain phenotype could be explained by the up-regulation of p11 that occurs in these mice, which could mask PDZD2 effects by increasing the amount of membrane associated Na_V_1.8 [44]. Interestingly, the assumption that Na_V_1.8 is solely expressed in DRG has been challenged by the discovery that both mouse and human atria and ventricles contain mRNA for Na_V_1.8 and significantly a mutation in the channel is linked to cardiac problems [6]. This mutation V1073A in the second intracellular loop of Na_V_1.8, is flanked by a putative phosphorylation site (RKDS) and a class I PDZ binding motif. As both PDZD2 and syntrophin-associated serine/threonine kinase (SASTK) have been shown to bind this intracellular loop [30] (Fig. 1), interaction with these molecules may be linked to the trafficking and electrophysiological properties of Na_V_1.8.

## 4 Contactin

Contactin is a glycosyl-phosphatidylinositol-anchored CAM protein expressed by neurons. Reports show that it co-immunoprecipitates with sodium channels from the brain and enhances Na_V_1.2 currents when co-expressed with β1 subunit in a heterologous system [21]. Also, the interaction between Contactin, β1 subunit, and Ankyrin is required to keep Na_V_1.2 at high density on the cell membrane [28]. Further to this, Contactin binds to Na_V_1.3, resulting in increased sodium peak currents without changing the electrophysiological properties of the channel [43]. In addition, Contactin regulates Na_V_1.8 and Na_V_1.9 mediated currents [37]. In Contactin knock-out mice, tetrodotoxin-resistant currents are decreased while tetrodotoxin-sensitive currents are unaltered in unmyelinated DRG neurons. In these animals the Na_V_1.8 activation curve was shifted to a more depolarised potential (i.e. a greater depolarisation is needed for activation) but no changes were apparent for Na_V_1.9. Contactin deficient mice also exhibit a reduction of Na_V_1.8 and Na_V_1.9 (but not of Na_V_1.6 and Na_V_1.7) immunoreactivity along unmyelinated axons in the sciatic nerve, suggesting a role of Contactin in regulating the surface expression of these channels.

## 5 PGE2-dependent trafficking to the plasma membrane

**Fig. 4:**
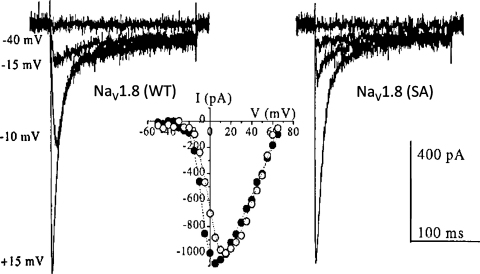
Comparison of voltage-dependence of current activation in wild-type Na_V_1.8 (WT) and the mutant Na_V_1.8 (SA) which lacks five PKA consensus phosphorylation sites within the intracellular loop between domain I and II. Representative currents and associated current–voltage plots from a wild-type Na_V_1.8-transfected cell and a cell transfected with mutant Na_V_1.8 (SA) are shown. The current–voltage plots shows decrease in peak current and right shift of voltage-dependence of activation in the mutant Na_V_1.8 expressed cells. Closed circle – Na_V_1.8 (WT); open circle – Na_V_1.8 (SA).

It is well established that during inflammatory conditions several compounds such as prostaglandin E2 (PGE2), adenosine triphosphate, Bradykinin, nerve growth factor and histamine activate intracellular cascades resulting in sensitisation of nerve terminals and lowered pain thresholds. Among these compounds, PGE2, a derivate of arachidonic acid, binds to its receptors, EP2 and EP4, which in turn activate intracellular Protein kinase A (PKA) (reviewed in Refs. [31,25]). PKA phosphorylates serine residues within the intracellular loop I of Na_V_1.8 and alters its gating properties, shifting its steady-state activation curve to a more negative potential [8] (Fig. 4). This change may account for an increase in Na_V_1.8 mediated sodium currents and higher cell excitability. However, increased trafficking of Na_V_1.8 to the cell membrane has recently been discovered to substantially contribute to this phenomenon [27]. It has been demonstrated that PGE2 directly promotes surface expression of Na_V_1.8 in a dose-dependent manner, and this effect can be blocked by cellular trafficking inhibitors. Forskolin, an activator of PKA, increases the Na_V_1.8 density on the membrane and mutation of the phosphorylation sites (RXXS) on Na_V_1.8 abolishes forskolin mediated effects. Interestingly, this effect of PKA phosphorylation is dependent on the ER retention motif RRR (whose sequence is shared with a phosphorylation site), but is independent of the masking effect of the β3 subunit mentioned above.

## 6 CAP-1A

Endocytosis is known to regulate the surface presentation of many membrane proteins. Sodium channels have been demonstrated to be selectively degraded in acidic/lysosomal vesicles upon infection of DRG neurons by herpes simplex virus [47]. Using the C-terminus of Na_V_1.8 as bait in yeast two-hybrid screens, CAP-1A (Clathrin-associated protein-1A) was identified as a binding partner [26]. CAP-1A is thought to bind to a conserved motif also present in other sodium channels such Na_V_1.2 [11] and act as an adaptor protein, linking voltage-gated sodium channels to clathrin, which is involved in coated vesicle assembly. Calmodulin binding to an IQ motif within the C-terminus has been proposed to disrupt CAP-1A binding and subsequently inhibit its cell surface removal by the endocytic pathway [7].

## 7 Local translation

There is a growing body of evidence suggesting that local translation, where transcription is distal from the final location of protein synthesis, is an important mechanism regulating many cellular functions. mRNA transportation, localisation and subsequent “local translation” is thought to be especially important in highly polarised cells, including neurons, where a rapid response to stimuli would be limited by long distance protein transport. In neuronal cells, it has been demonstrated to play a role in axonal growth [29], long-term potentiation in the brain [5], synaptic plasticity [51] and to modulate pain threshold [17]. At present, “local synthesis” of transmembrane proteins is controversial as their synthesis and post-translational modifications (e.g. glycosylation) requires both ER and Golgi at the location of synthesis, although tantalising evidence suggests that some membrane proteins can be synthesised locally such as GluR and IP3RI, where these membrane proteins are expressed along dendrites and at the synapses respectively [46,15].

A recent study has investigated the potential involvement of local translation on Na_V_1.8 protein changes in a model on neuropathic pain [50]. Upon sciatic nerve entrapment it has been found that compound action potential and Na_V_1.8 immunoreactivity increased in the treated nerve. Concomitant with these findings, Na_V_1.8 mRNA level was up-regulated in the sciatic nerve but remained unchanged in the DRG, while no changes were detected for the mRNAs of other channel isoforms. It has been proposed that specific and enhanced transport of Na_V_1.8 mRNA into the axons may account for this up-regulation and that local mRNA accumulation could contribute to increased axonal Na_V_1.8 protein levels. Recent evidence using rapamycin inhibition of mTOR suggests that, in DRG, Aδ but not C-fibre neurons contain the machinery to carry out local translation [17]. Interestingly, a small subset of C-fibres may indeed be capable of local translation, although more work needs to be carried out to confirm this result. A recent study measuring the axonal transport velocity of Kv1.2 recorded a rate of 0.8 μm/s [14]. Assuming this velocity is similar for sodium channels, the transport of molecules to the extreme ends of the axon may require many days. Plasma membrane inserted sodium channels exhibit a half-life of around 50 h and those within the intracellular pool significantly less (∼30 h) [39]. These values place an upper limit on the distance the channels could diffuse or be actively transported to their destination.

Because of its involvement in pain pathways and tissue-specific localisation, Na_V_1.8 has been one of the best studied sodium channels and expected to be a good target for the treatment of chronic pain. Although many attempts have been made to develop specific blockers for Na_V_1.8 [10,16], the development of type-specific blockers of individual sodium channels has remained challenging due to the similarities among the sodium channels. Successful use of gabapentin/pregabalin for the treatment of neuropathic pain provided a new route to block channel activities, i.e. targeting accessory subunits to inhibit the localisation of calcium channels to the plasma membrane and/or to alter the activation kinetics [49]. The trafficking mechanisms of Na_V_1.8 discussed above may provide such novel targets for the treatment of chronic pain.

## Figures and Tables

Reproduced, with permission, from Ref. [32].

Reproduced, with permission, from Ref. [44].

Reproduced, with permission, from Ref. [8].
